# MicroRNA-26a/b-5p promotes myocardial infarction-induced cell death by downregulating cytochrome c oxidase 5a

**DOI:** 10.1038/s12276-021-00665-0

**Published:** 2021-09-13

**Authors:** Seung Eun Jung, Sang Woo Kim, Seongtae Jeong, Hanbyeol Moon, Won Seok Choi, Soyeon Lim, Seahyoung Lee, Ki-Chul Hwang, Jung-Won Choi

**Affiliations:** 1 Institute for Bio-Medical Convergence, College of Medicine, Catholic Kwandong University, Gangneung-si, Gangwon-do 25601 Republic of Korea; 2 International St. Mary’s Hospital, Catholic Kwandong University, Incheon Metropolitan City, 22711 Republic of Korea; 3 Department of Integrated Omics for Biomedical Sciences, Graduate School, Yonsei University, Seoul, 03722 Republic of Korea

**Keywords:** Protein-protein interaction networks, Predictive markers

## Abstract

Myocardial infarction (MI) damage induces various types of cell death, and persistent ischemia causes cardiac contractile decline. An effective therapeutic strategy is needed to reduce myocardial cell death and induce cardiac recovery. Therefore, studies on molecular and genetic biomarkers of MI, such as microRNAs (miRs), have recently been increasing and attracting attention due to the ideal characteristics of miRs. The aim of the present study was to discover novel causative factors of MI using multiomics-based functional experiments. Through proteomic, MALDI-TOF-MS, RNA sequencing, and network analyses of myocardial infarcted rat hearts and in vitro functional analyses of myocardial cells, we found that cytochrome c oxidase subunit 5a (Cox5a) expression is noticeably decreased in myocardial infarcted rat hearts and myocardial cells under hypoxic conditions, regulates other identified proteins and is closely related to hypoxia-induced cell death. Moreover, using in silico and in vitro analyses, we found that miR-26a-5p and miR-26b-5p (miR-26a/b-5p) may directly modulate Cox5a, which regulates hypoxia-related cell death. The results of this study elucidate the direct molecular mechanisms linking miR-26a/b-5p and Cox5a in cell death induced by oxygen tension, which may contribute to the identification of new therapeutic targets to modulate cardiac function under physiological and pathological conditions.

## Heart attack: Possible biomarker may lead to cell repair treatments

Monitoring the activity of two microRNAs, small non-coding RNAs, may provide a useful biomarker for heart attack prognosis and inform novel treatments for repairing heart cells. Ki-Chul Hwang and Jung-Won Choi at the Catholic Kwandong University in Gangwon-do, South Korea, and co-workers examined potential causative factors for heart attacks by exploring the activity of microRNAs in rat models and heart cell cultures. They found that levels of a key protein involved in maintaining mitochondrial function and energy metabolism, cytochrome c oxidase subunit 5a (Cox5a), were significantly decreased in heart cells during oxygen deprivation. Further, they identified two microRNAs that acted to inhibit Cox5a after a heart attack. Suppressing these two microRNAs could boost Cox5a activity and limit cell death, although the authors urge caution because microRNAs also play physiological roles in the body.

## Introduction

Myocardial infarction (MI) is a type of coronary artery disease (CAD) caused by the death of myocardial cells and damage to cardiac muscle due to occlusion of coronary arteries^1,2^. CAD is one of the major causes of morbidity and mortality and leads to more than seven million deaths worldwide annually^1^. Once the intravascular oxygen supply is blocked, MI is triggered within 20 min, and complete cell death occurs within a few hours^1,3^. Various studies have shown that MI damage induces various types of cell death, including necrosis, apoptosis, and autophagy^4,5^. In a previous study, we determined the presence of necroptotic and apoptotic cell death in association with cardiac ischemic injury and the inhibitory effects of microRNAs (miRs) on cell death^6^. Persistent ischemia causes cardiac contractile decline by decreasing the proliferation and dysfunction of cardiomyocytes^1,3^. Currently, treatment for MI is focused on reducing the severity of MI rather than on restoring cardiac tissue^7^. Therefore, a therapeutic strategy is needed to reduce myocardial cell death and induce cardiac recovery^1^.

Identifying and assessing cardiac biomarkers can be useful for this therapeutic strategy in MI. Cardiac biomarkers have been used for the diagnosis of MI and determination of prognosis after MI treatment, and include inflammatory (c-reactive protein, pentraxin-3, interleukin-6, etc.), plaque destabilization (pregnancy-associated plasma protein A, myeloperoxidase, tumor necrosis factor α, etc.), and myocardial necrosis (troponin, myoglobin, growth-differentiation factor-15, etc.) markers^8,9^. However, these markers have several limitations, including insufficient specificity, varying times of detection, and difficulty of therapeutic monitoring^10^. Therefore, studies on molecular and genetic biomarkers for MI, such as miRs, have recently been increasing and attracting attention due to the ideal characteristics of these molecules^10^. They are easily accessible via noninvasive methods, quite stable in various biological fluids and sensitive and specific to MI pathology^10^.

miRs are small noncoding RNAs that control gene expression by binding to mRNA and contribute to all cellular processes^11–13^. Some miRs are involved in the pathophysiological pathway of MI and can either promote or block cell death in MI models^3^. Recent studies have shown that some miRs control apoptosis, autophagy, and necroptosis by regulating key factors under pathophysiological conditions^14^, but several miRs can regulate cardiomyocyte proliferation, angiogenesis, progenitor, or stem cell repair and cell-to-cell communication^3^. In other words, miRs can play either an adverse or protective regulatory role. For example, miR-15, miR-195, and miR-34 impair cardiomyocytes, while miR-24, miR-214, and miR-7a/b promote cardiomyocyte survival^15–19^. Furthermore, miRs can synergistically enhance therapeutic efficiency by reducing side effects and preventing drug resistance^20^. Accumulating evidence shows that miRs function in MI via their effects on myocardial cell death and cardiomyocyte regeneration pathways. These findings have increased our understanding of the miR-regulated signaling pathways in MI and cardiac pathogenesis. Evidence of the potential of miRs as new biomarkers for MI diagnosis and prognosis determination continues to increase.

The present study aimed to discover novel causative factors of MI using multiomics-based functional studies. Through proteomics, matrix-assisted laser desorption/ionization time-of-flight mass spectrometry (MALDI-TOF-MS), RNA sequencing and network analyses of myocardial infarcted rat hearts and in vitro functional studies using myocardial cells, we found that cytochrome c oxidase subunit 5a (Cox5a) expression is noticeably decreased in myocardial infarcted rat hearts and myocardial cells under hypoxic conditions, regulates other identified proteins and is closely related to cell death induced by a decreased oxygen level. In addition, we used in silico and in vitro analyses and found that miR-26a-5p and miR-26b-5p (miR-26a/b-5p) may modulate Cox5a, which regulates hypoxia-induced cell death. The results of this study elucidate the molecular mechanisms linking miR-26a/b-5p and Cox5a in cell death induced by oxygen tension, which may contribute to the establishment of new therapeutic targets to modulate cardiac function under physiological and pathological conditions.

## Materials and methods

### Establishment of MI rat models

All experimental procedures for animal studies were approved by the Committee for the Care and Use of Laboratory Animals of Catholic Kwandong University College of Medicine (CKU01-2018-003), and performed following the Committee’s Guidelines and Regulations for Animal Care. Eight-week-old male Sprague–Dawley rats (250 ± 30 g, KOATECH, Gyeonggi‐do, Republic of Korea) were ventilated via the trachea using a ventilator (Harvard Apparatus, Holliston, MA, USA) after anesthetization with Zoletil (30 mg/kg; Virbac, France) and xylazine (10 mg/kg, Bayer Korea, Ansan, Gyeonggi-do, Republic of Korea), and MI was surgically induced^21^. MI was induced by surgical occlusion of the left anterior descending coronary artery via ligation using a 6-0 Prolene suture (Ethicon, Diegem, Belgium) for 24 h. Rats were sacrificed the following day to obtain cardiac tissue.

### 2-DE analysis, image acquisition, and data analysis

Proteins were isolated from rat hearts using TRIzol reagent (Life Technologies, Frederick, Maryland, USA), and the protein content was determined using the Bradford protein assay (Bio-Rad, Hercules, CA, USA). Two-dimensional electrophoresis (2-DE) analysis was performed in triplicate using protein samples (50 µg per gel) from control rats (*n* = 3) and MI rats (*n* = 3) according to optimized methods described in our previous studies^22,23^. Gels were imaged in a UMAX PowerLook 1120 instrument (Maxium Technologies, Akron, OH, USA), and comparison of images between groups was carried out using modified ImageMaster 2D software V4.95 (GE Healthcare, Buckinghamshire, UK) according to the manufacturer’s instructions, and the commonly used methods reported in our previous studies^22,23^.

### PMF analysis

MALDI-TOF analysis (Microflex LRF 20, Bruker Daltonics) was performed as described by Fernandez et al.^24^. The search program MASCOT (Mascot Server 2.3), developed by Matrixscience (http://www.matrixscience.com), was used, and the MASCOT probability-based molecular weight search (MOWSE) score was calculated for PMF analysis. The PMF acceptance criteria were based on probability scoring as follows: −10*Log (*P*), where *P* is the probability that an observed match is a random event, and a score higher than 62 is significant (*p* < 0.05).

### Network analysis

For gene and protein network analyses, proteins identified by proteomic analysis (Table 1) were analyzed using STRING v11.0 (http://string-db.org). Associations between differentially expressed genes/proteins and broadly defined molecular networks were combined and visualized using the STRING database. Using the web interface, we predicted gene/protein interactions and their interacting partner genes/proteins identified in this study.

**Table 1:** List of identified proteins in rat heart by peptide mass fingerprinting (PMF) analysis.

Spot ID	Description	Gene name	Acc. no.^a^	Monoisotopic mass (Mr)	Calculated PI	Score^b^	Fold changes (MI/CON)	*p* value^c^
1	Peroxiredoxin-2	Prdx2	NP_058865.1	21,941	4.41	62	0.50	0.000
5	NADH dehydrogenase [ubiquinone] iron-sulfur protein 8	Ndufs8	NP_001099792.1	24,411	5.87	66	0.31	0.001
6	ATP synthase subunit d	Atp5pd	NP_062256.1	18,809	5.45	100	0.03	0.009
9	Cytochrome c oxidase subunit 5a precursor	Cox5a	NP_665726.1	16,347	6.08	66	0.47	0.009
10	Heat shock protein β-6	Hspb6	NP_620242.1	17,551	6.05	155	0.09	0.018
13	NADH dehydrogenase [ubiquinone] flavoprotein 2 precursor	Mdufv2	NP_112326.1	27,703	6.23	113	0.61	0.012
14	Adenine phosphoribosyltransferase	Aprt	NP_001013079.1	19,761	6.17	145	0.39	0.014
15	V-set and transmembrane domain-containing protein 2A	Vstm2a	XP_008765103.1	24,560	6.21	88	0.40	0.010
16	Annexin A5	Anxa5	NP_037264.1	35,779	4.99	296	0.41	0.018
19	Mitochondrial aldehyde dehydrogenase precursor	Aldh	AAS75814.1	56,079	6.69	230	1.38	0.038
20	Fatty acid-binding protein	Fabp3	NP_077076.1	14,766	5.9	124	0.72	0.049
24	α-enolas	Eno1	XP_006239506.1	47,627	5.9	169	1.27	0.014
25	Dihydrolipoamide S-acetyltransferase	Dlat	pir| |I55976	48,117	5.97	116	0.68	0.003
29	NADH dehydrogenase (ubiquinone) 1α subcomplex 10 precursor	Ndufa10	NP_955789.2	40,753	7.64	183	0.18	0.082
30	Cytochrome c oxidase subunit 5a precursor	Cox5a	NP_665726.1	16,347	6.08	157	0.80	0.043
32	NADH dehydrogenase [ubiquinone] iron-sulfur protein 3	Ndufs3	NP_001099959.1	30,379	7.07	182	0.16	0.081
33	NADH dehydrogenase (ubiquinone) Fe-S protein 1	Ndufs1	EDL98902.1	74,362	5.74	204	0.40	0.011
34	Vinculin	Vcl	EDL86257.1	124,126	5.54	107	0.29	0.027
35	Pyruvate dehydrogenase E1 component subunit β precursor	Pdhb	NP_001007621.1	39,299	6.2	71	0.39	0.057
41	Hypoxanthine-guanine phosporibosyltransferase	LOC103689983	AAA41351.1	18,043	8.9	110	0.41	0.084
43	3-mercaptopyruvate sulfurtransferase	Mpst	NP_620198.1	33,205	5.88	123	0.38	0.033
43	Annexin A3	Anxa3	EDL99639.1	36,744	5.72	64	0.38	0.033
44	Myosin light chain 3	Myl3	NP_036738.1	22,256	5.03	121	0.65	0.020

*CON* control rat group, *MI* rat group with myocardial infarction.

^a^Acc. no. is the NCBIprot database accession number.

^b^Protein score equals −10*Log (*P*), where *P* is the probability that the observed match is a random event. Protein scores greater than 62 are significant (*p* < 0.05).

^c^Significant differences between CON and MI groups were determined via ANOVA.

### Total RNA sequencing and data analyses

Total RNA was isolated using TRIzol reagent, RNA quality was assessed with an Agilent 2100 bioanalyzer using an RNA 6000 Nano Chip (Agilent Technologies, Amstelveen, The Netherlands), and RNA quantification was performed using an ND-2000 spectrophotometer (Thermo Fisher Scientific, Wilmington, DE, USA). Libraries were prepared using a SMARTer Stranded RNA-Seq Kit (TaKaRa Bio, Mountain View, CA, USA), and ribosomal RNA was removed using a RiboCop rRNA Depletion Kit (Lexogen, Vienna, Austria). High-throughput sequencing was performed with 100 bp paired-end reads using the HiSeq 2500 system (Illumina, San Diego, CA, USA). Total RNA sequencing reads were mapped using TopHat software^25^, and data mining and graphical visualization were performed using ExDEGA (e-Biogen, Seoul, Republic of Korea).

### Preparation of normoxia-conditioned and hypoxia-conditioned H9c2 cells

H9c2 cells were obtained from the Seoul Korean Cell Line Bank (Seoul, Republic of Korea) and cultured in Dulbecco’s modified Eagle’s medium (DMEM; HyClone, Logan, UT, USA) supplemented with 10% fetal bovine serum (FBS; HyClone) and 1% penicillin/streptomycin in a humidified atmosphere with 5% CO_2_ at 37 °C. H9c2 cells were incubated with serum-free medium (SFM) under normoxic or hypoxic conditions for 3, 6, or 12 h. Under hypoxic conditions, cells were incubated at 37 °C in 5% CO_2_, 5% H_2,_ and 0.5% O_2_ in a chamber with an anaerobic atmosphere system (Technomart, Seoul, Republic of Korea). The cells were harvested after the 3, 6, or 12 h of incubation period.

### Mitochondrial isolation

For mitochondrial isolation, H9c2 cells were cultured in SFM under normoxic or hypoxic conditions and harvested after a 12 h of incubation period. Mitochondrial pellets were separated using a mitochondrial isolation kit (Thermo Fisher Scientific; Rockford, IL, USA) in accordance with the suggested instructions. In brief, mitochondrial isolation was performed at 4 °C by differential velocity centrifugation (700×*g* for 10 min; 12,000×*g* for 5 min) steps using the mitochondrial isolation kit. The final mitochondrial pellet was lysed with 2% CHAPS (Bio Basic, Markham, Canada) in Tris-buffered saline (TBS; Biosesang; Seongnam-si, Gyeonggi-do, Republic of Korea). The lysate was measured using a BCA assay kit (Thermo Fisher Scientific).

### Transfection with Cox5a siRNA, miRs, and anti-miRs

Cells were seeded at a density of 5 × 10^3^ cells/well in a 96-well plate or 1.5 × 10^5^ cells/well in a 6-well plate before transfection, and were then transiently transfected with siRNAs, miRs, or anti-miRs (1 pmol/well in the 96-well plate and 25 pmol/well in the 6-well plate) using Lipofectamine RNAiMAX Reagent (Invitrogen, Carlsbad, CA, USA) (0.3 μL/well in the 96-well plate and 7.5 μL/well in the 6-well plate). Transient knockdown with Cox5a Commercial AccuTarget siRNAs (Bioneer, Daejeon, Republic of Korea), which are target-specific siRNAs (Entrez Gene ID 252934-1: sense (5′-3′), GAGUUGCGUAAAGGGAUGA (dTdT) and antisense (5′-3′), UCAUCCCUUUACGCAACUC (dTdT); 252934-2: sense (5′-3′), GAGUUGCGUAAAGGGAUGA (dTdT) and antisense (5′-3′), UCAUCCCUUUACGCAACUC (dTdT)), was performed to knock down Cox5a gene expression, and AccuTarget Negative Control siRNA (Bioneer) was also used. In addition, the sequences of the rno-miR-26a/b-5p mimic and inhibitor (Genolution, Seoul, Republic of Korea) were as follows: mature rno-miR-26a-5p—MIMAT0000796: mimic (5′-3′), UUCAAGUAAUCCAGGAUAGGCU and inhibitor (5’-3’), AAGUUCAUUAGGUCCUAUCCGA; and mature rno-miR-26b-5p—MIMAT0000797: mimic (5′-3′), UCAAGUAAUUCAGGAUAGGU and inhibitor (5′-3′), AAGUUCAUUAAGUCCUAUCCA. AccuTarget miRNA negative controls (Bioneer) were used as negative controls in miR experiments. The miR mimic and inhibitor negative controls were used separately, and the two negative controls were used together for luciferase assays.

### Immunoblot analysis

Protein isolation and immunoblot analysis were performed as described previously^22,26^. Briefly, cells were lysed in RIPA buffer (Thermo Fisher Scientific) mixed with a phosphatase inhibitor (Sigma-Aldrich, St. Louis, MO, USA), protease inhibitor (Sigma-Aldrich), and proteasome inhibitor (MG132; Abcam, Cambridge, UK). Proteins were detected using SDS-PAGE and were then electrotransferred to polyvinylidene difluoride (PVDF; Sigma-Aldrich) membranes. Next, the membranes were blocked with 5% skim milk (BD Difco; Sparks, MD, USA) for 1 h and incubated with primary antibodies (Santa Cruz, Dallas, TX, USA) overnight at 4 °C. After washing, the membranes were stained with horseradish peroxidase (HRP)-conjugated secondary antibodies (Enzo Life Sciences, Lausen, Switzerland) for 1 h. In addition, the blots were developed with enhanced chemiluminescence reagents (ECL Western blotting Detection Kit, GE Healthcare), and the band intensities were analyzed using ImageJ software (NIH).

### Immunofluorescence analysis

Cells were grown on cell culture slides (SPL, Pocheon, Gyeonggi-do, Republic of Korea) and were then fixed with 4% formaldehyde (Biosesang). After one wash with phosphate-buffered saline (PBS), the slides were subjected to antigen retrieval in sodium citrate buffer for 10 min at 95 °C and were then incubated for permeabilization in 0.2% Triton X-100 (Sigma-Aldrich) for 10 min at room temperature. The slides were then blocked with 0.5% BSA for 1 h. After washing with PBS, the slides were incubated with an anti-Cox5a antibody (1:200 dilution) overnight at 4 °C. The following morning, the slides were incubated with a rhodamine-conjugated mouse secondary antibody (1:1000 dilution). DAPI (Sigma-Aldrich) was used to stain cell nuclei. The prepared slides were observed using an LSM700 confocal laser scanning microscope (Carl Zeiss, Oberkochen, Germany). Images were acquired using Zen Black or Blue software (Carl Zeiss).

### RNA isolation, reverse transcription (RT)-PCR, and qPCR analyses

Total RNA was isolated from H9c2 cells and primary cardiomyocytes using an Easy-Spin Total RNA Extraction Kit (iNtRON Biotechnology, Seongnam-si, Gyeonggi-do, Republic of Korea) according to the manufacturer’s instructions. mRNA transcript levels in total RNA samples were quantitatively determined using a Maxime RT PreMix Kit (iNtRON Biotechnology). The transcript level of each gene was quantitatively determined by qPCR using an Applied Biosystems StepOnePlus Real-Time PCR System (Foster City, CA, USA) with a SYBR Green dye system (SYBR Premix Ex Taq, Tli RNase Plus and ROX reference dye; TaKaRa Bio; Foster City, CA, USA). All values are shown as normalized target gene expression levels (fold change; 2^∆∆Ct^) with reference to Gapdh transcript levels. Primers were designed using Primer3 and BLAST, and the primer set sequences were as follows: Cox5a sense (5′-3′), ATGCTCGCTGGGTGACATAC and antisense (5′-3′), ATGCCCTCAAAGCAGCATCA; Gapdh sense (5′-3′), TCTCTGCTCCTCCCTGTTCTA and antisense (5′-3′), GGTAACCAGGCGTCCGATAC. miR transcript levels were quantitatively determined using RT (TaqMan MicroRNA Reverse Transcription Kit; Applied Biosystems, Waltham, MA, USA), and the miR (miR-26a-5p (Assay ID, rno481013_mir), miR-26b-5p (Assay ID, rno481461_mir) and U6 control (Assay ID, 001973) transcripts were quantified using TaqMan miRNA assays (Thermo Fisher Scientific) according to the manufacturer’s instructions. The threshold cycle (Ct) value of each miR was normalized to that of the U6 control (ΔCt value), and the relative differences in the expression levels of miRs between groups (ΔΔCt) were calculated and are presented as fold change (2^−ΔΔCt^) values.

### Cell viability and cytotoxicity assays

To assess cell death induced by Cox5a knockdown in H9c2 cells and primary cardiomyocytes, knockdown cells were exposed to normoxia or hypoxic stress for 12 h. The supernatants were subjected to a lactate dehydrogenase (LDH) assay using a Cytotoxicity Detection Kit (TaKaRa Bio, Nojihigashi, Kusatsu, Shiga, Japan), and the viability of knockdown cells was measured using Ez-Cytox (DOGEN, Seoul, Republic of Korea) according to the manufacturer’s instructions.

### Luciferase assay

The whole 3′ UTR (150 bp) of Cox5a (Accession: NM_145783.1) was cloned into pEZX-MT05 (8713 bp; GeneCopoeia, Rockville, MD, USA), and the linker contained two different enzyme sites at the 5′ and 3′ ends of the whole 3′ UTR: AsiS1-Cox5a-EcoR1 and Xho1-Cox5a-Spe1. Ampicillin was applied as the antibiotic, and neomycin was used as the stable selection marker. The reporter genes were Gluc and SeAp, and the SV40 promoter was used. H9c2 cells and primary cardiomyocytes were cotransfected with empty vector or plasmids containing the whole 3′ UTRs of Cox5a and miR-26a-5p, miR-26b-5p or the negative control miR using Lipofectamine RNAiMax Reagent (Invitrogen). After 24 and 48 h of incubation, luciferase activity was measured using a Dual-Luciferase Assay Kit (Promega; Madison, WI, USA) according to the manufacturer’s instructions. Renilla luciferase (Promega) was used to normalize the cell number and transfection efficiency.

### Isolation of primary cardiomyocytes from neonatal rat hearts

Ventricles isolated from 1-day-old to 2-day-old Sprague-Dawley rats (KOATECH) were washed with cold PBS and minced with fine scissors into pieces of 1–3 mm^[327^. Then, the ventricle pieces were incubated in 2 mg/mL collagenase type II (Worthington Biochemical, Lakewood, NJ, USA) at 37 °C for 7 min. The supernatant was carefully transferred to a new tube and centrifuged at 1600 rpm for 3 min. The cell pellet was resuspended in culture medium and incubated at 37 °C. These steps were repeated 8–10 times using the remaining ventricle pieces, and all the cells were collected. Harvested cells were preplated on an uncoated culture dish to reduce fibroblast contamination, and nonadherent cardiac myocytes were then plated on 1.5% gelatin (Sigma-Aldrich)-coated plates. Cells were cultured in alpha-minimum essential medium (α-MEM, HyClone) containing 10% FBS in a humidified atmosphere with 5% CO_2_ at 37 °C.

### Statistical analysis

All data were compared via one-way analysis of variance (ANOVA) using Statistical Package for the Social Sciences (SPSS, version 14.0K) software. Data are expressed as the means ± SEMs. Differences between groups were considered significant at *p* < 0.05, as determined by the protected least-significant difference (LSD) test when ANOVA indicated an overall significant treatment effect (*p* < 0.05).

## Results

### Exploration of the causative factors of MI

**Fig. 1:**
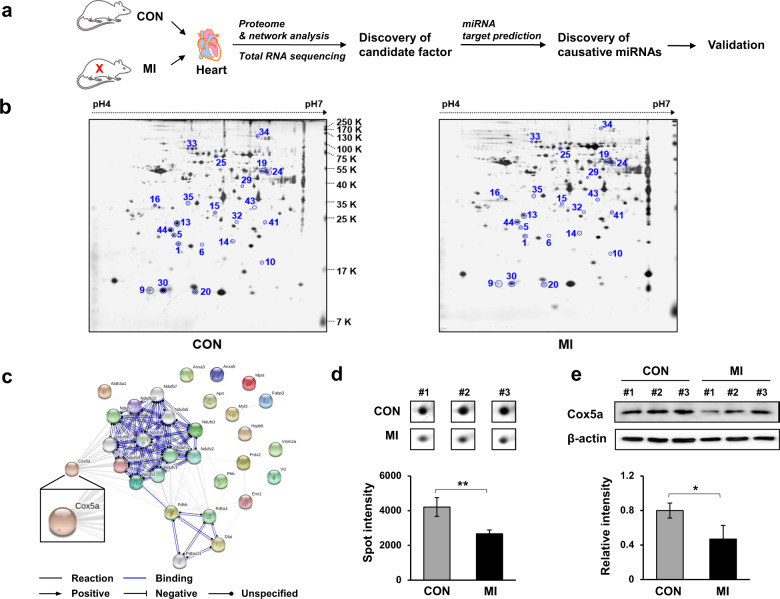
Discovery of novel MI-related factors using proteomic and network analyses. **a** Schematic showing the experimental procedure of our study. **b** Representative silver-stained 2-DE gel images and **c** networks generated by STRING database analysis using differentially expressed factors in the hearts of control and MI rats. **d** Magnified images of the 2-DE gels and **e** validated results for cytochrome c oxidase 5a (Cox5a) protein expression in each group by immunoblot analysis. 2-DE experiments were performed in triplicate for each individual, and immunoblot experiments were carried out twice independently. Spot and band intensities were measured as area densities and analyzed using ImageJ software. The relative intensity level of an immunoblot band indicates the target protein level normalized to the β-actin level. Significant differences between groups were determined via ANOVA, with *p* values indicated as *p < 0.05 and **p < 0.01. CON control rat group, MI myocardial infarction rat group. *n* = 3 rats per group.

**Fig. 2:**
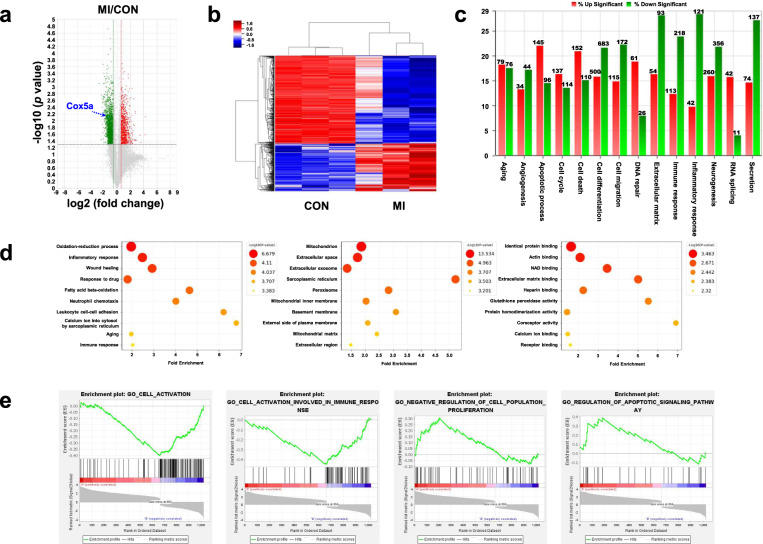
Transcriptome analysis of MI using total RNA sequencing analysis. **a** Volcano plot and **b** hierarchical clustering heatmap of differentially expressed genes in the hearts of control and MI rats. **c** Significantly expressed genes and **d** fold enrichment of genes determined by Gene Ontology analysis with DAVID. **e** GSEA of up/downregulated genes revealed molecular pathways related to cell activation, cell activation involved in the immune response, negative regulation of cell population proliferation, and regulation of the apoptotic signaling pathway. The green dots in the volcano plot indicate genes with decreased expression, and the red dots indicate genes with increased expression in MI compared with CON. Significant differences between groups were determined via ANOVA, with *p* values indicated as *p < 0.05 and **p < 0.01. CON control rat group, MI myocardial infarction rat group. *n* = 3 rats per group.

**Fig. 3:**
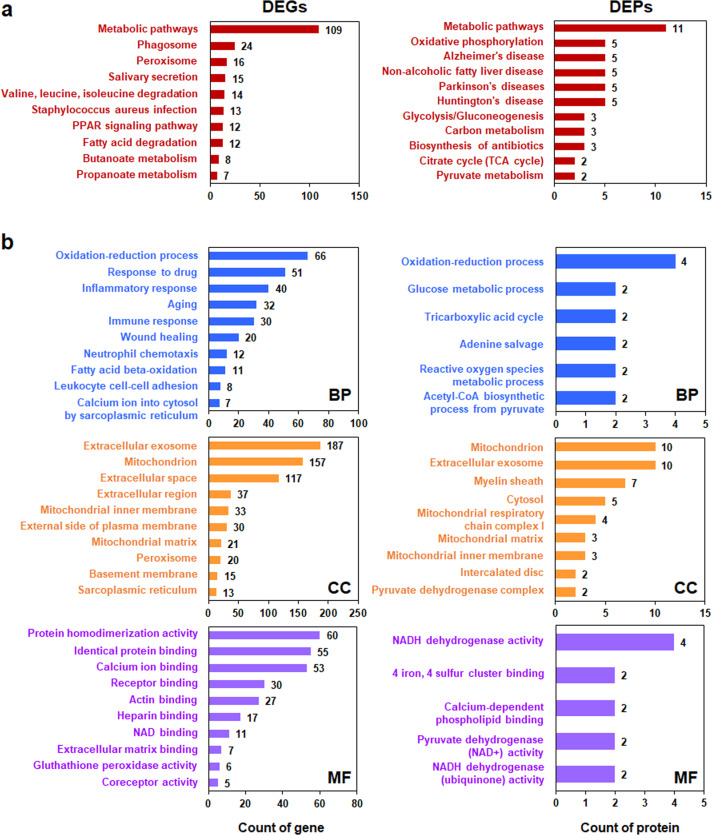
Kyoto Encyclopedia of Genes and Genomes (KEGG, a) and Gene Ontology (GO, b) analyses of differentially expressed genes (DEGs) and differentially expressed proteins (DEPs). The DEGs and DEPs were grouped into biological process (BP), cellular component (CC), and molecular function (MF) categories. The number of genes or proteins in each category is plotted on the *x*-axis.

To discover novel causative factors of MI, 2-DE-based proteomic analysis combined with MALDI-TOF-MS and network analyses were performed using the hearts of MI rats (Fig. 1a). Rats were randomly divided into two groups of three rats each. Rats in one group (control rats (*n* = 3)) were sutured after opening of the thoracic cavity without intervention, whereas rats in the other group (MI group (*n* = 3)) were subjected to surgically induced MI via occlusion of a coronary artery. 2-DE-based proteomic experiments were carried out using isolated cardiac tissue to investigate proteins differentially expressed between the two groups, and nearly 240 individual spots were detected, with masses ranging from 7 to 250 kDa between pH 4 and 7 (Fig. 1b). Forty-three spots showing significant differential expression between groups were identified among the 240 spots examined, and 22 different proteins were identified by peptide mass fingerprinting (PMF) (Table 1). Most of the proteins in the MI group were downregulated compared with those in the control group, except for two proteins—mitochondrial aldehyde dehydrogenase precursor and α-enolase (Table 1). In addition, networks were generated via STRING database analysis using the genes/proteins upregulated or downregulated by MI to predict the interaction of factors with differential expression between groups (Fig. 1c). Interestingly, we found that Cox5a was closely associated with other discovered factors (Fig. 1c). Therefore, we selected Cox5a as a candidate factor involved in the pathophysiology of MI. In the 2-DE-based proteomic analysis, Cox5a was significantly decreased in the MI group compared with the control group (Fig. 1d), and this result was reconfirmed by immunoblot analysis (Fig. 1e). Through total RNA sequencing, we found that the Cox5a transcript in the MI group was markedly downregulated compared with that in the control group, consistent with the proteomic studies (Fig. 2a). A total of 6569 genes were differentially expressed in MI rat hearts (fold change ≥2, log2 normalized read count ≥4 to minimize false counts, *p* < 0.05). The hierarchical clustering heatmap in Fig. 2b shows the up/downregulated genes. Gene Ontology analysis of differentially expressed genes is shown in Fig. 2c. A total of 241 apoptotic protein-associated genes (145 upregulated and 96 downregulated) were differentially expressed ≥2-fold in MI rat hearts (*p* < 0.05) compared with normal rat hearts. Furthermore, the total set of differentially expressed genes was subjected to Gene Ontology analysis using DAVID (Fig. 2d). Highly relevant and significantly enriched terms identified by biological process analysis included “oxidation-reduction process”, “inflammatory response”, and “wound healing”. Enriched cellular component terms included a number of significant and relevant terms highlighting target gene associations with “mitochondria”, “extracellular space”, and “extracellular exosomes”. Enriched molecular function terms were largely related to identical protein binding, actin binding, and NAD binding. Interestingly, our gene set enrichment analysis (GSEA) revealed that “cell activation” and “cell activation in immune response” were substantially downregulated in MI rat hearts, while “negative regulation of cell population/proliferation” and “regulation of apoptotic signaling pathway” were upregulated in ischemic rat hearts (Fig. 2e and Supplementary Fig. 1). To better understand MI using multiomics-based functional studies, we combined transcriptomic and proteomic analysis data for more in-depth analysis via Kyoto Encyclopedia of Genes and Genomes (KEGG) pathway and Gene Ontology (GO) term enrichment analyses (Fig. 3). In the KEGG pathway enrichment analysis, all the differentially expressed genes (DEGs) and differentially expressed proteins (DEPs) were significantly enriched in metabolic pathways (Fig. 3a). In the biological process category, all the DEGs and DEPs were mainly involved in the oxidation-reduction process (Fig. 3b). In the cellular component category, all the DEGs and DEPs were mainly involved in mitochondria and extracellular exosomes (Fig. 3b). The GO terms in the biological process and cellular component categories most enriched with DEGs were the same as those most enriched with DEPs. The most significantly enriched pathway was “oxidation-reduction process” in both the transcriptomic and proteomic analysis results, indicating that oxidative stress and redox signaling might play a pivotal role in hearts with MI.

### Differential expression of Cox5a induced by oxygen tension in H9c2 cells

**Fig. 4:**
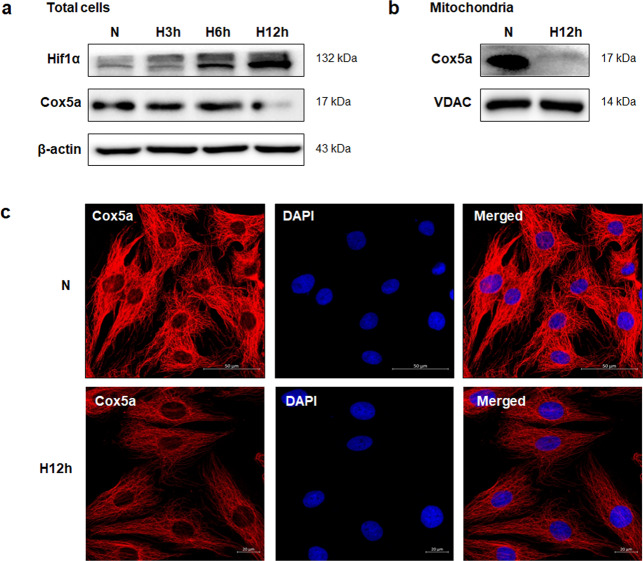
Cox5a expression in H9c2 cells under normoxic and hypoxic conditions. **a** Changes in the Cox5a level in total cells according to the hypoxic environment exposure time (3, 6, and 12 h) and **b** the mitochondrial Cox5a expression level 12 h after exposure to hypoxia were measured via immunoblot analysis; β-actin and VDAC were used as internal controls in total cells and mitochondria, respectively. **c** Changes in Cox5a expression after 12 h of incubation under normoxic and hypoxic conditions were investigated using immunocytochemical staining. Nuclei were stained with DAPI. Scale bar = 50 μm. The data are representative of two independent experiments.

We found decreased Cox5a expression in the MI rat model (Fig. 1) and tried to predict the functional contribution of Cox5a to MI in vitro. In brief, changes in Cox5a expression were investigated under a low oxygen environment in H9c2 cells, which are myoblasts from the rat heart/myocardium. Consequently, the Cox5a level was decreased the most when H9c2 cells were exposed to a hypoxic environment for 12 h (Fig. 4a), and this result was reaffirmed in isolated mitochondria (Fig. 4b). In addition, downregulation of Cox5a by hypoxic stress in H9c2 cells was validated using immunofluorescence staining (Fig. 4c).

### Effects of Cox5a knockdown on cell death induced by low oxygen tension in H9c2 cells

**Fig. 5:**
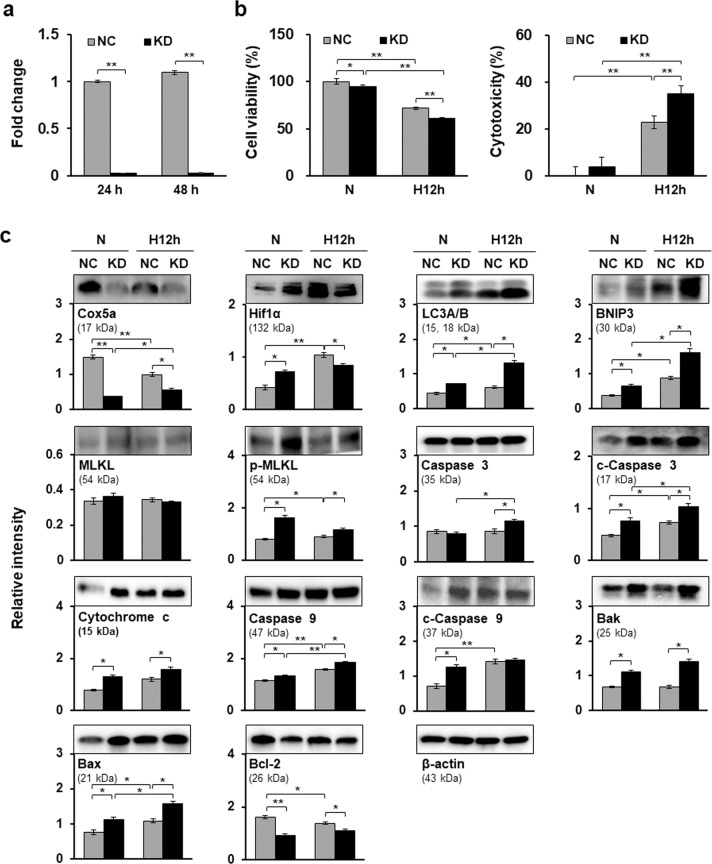
Effects of Cox5a knockdown on hypoxia-mediated cell death in H9c2 cells. **a** Cox5a knockdown via siRNA transfection was validated using qRT-PCR. All qRT-PCR data are shown as the target gene expression level normalized to the Gapdh expression level, and the data are representative of two independent experiments. **b** Changes in cell viability (left) and cytotoxicity (right) in H9c2 cells with Cox5a knockdown under normoxic and hypoxic conditions were determined by a WST-based cell viability assay and an LDH-based cytotoxicity assay, respectively. Experiments were performed in triplicate, and the data are representative of two independent experiments. **c** Cell death-related protein levels after Cox5a knockdown were measured using immunoblot analysis. Band intensities were measured as area densities and analyzed using ImageJ software. Relative intensity levels indicate the protein level normalized to the β-actin level. Immunoblot experiments were performed twice independently. Significant differences between groups were determined via ANOVA, with *p* values indicated as *p < 0.05 and **p < 0.01. N cells under normoxic conditions, H12h cells exposed to hypoxia for 12 h, NC negative control cells, KD Cox5a-knockdown cells.

To investigate the functions of Cox5a under low oxygen conditions, Cox5a knockdown was performed via siRNA transfection of H9c2 cells, and we verified that Cox5a expression was knocked down by approximately 97% after 24 and 48 h of siRNA transfection (Fig. 5a). After 24 h of siRNA transfection, H9c2 cells were exposed to normoxic or hypoxic conditions for 12 h. Then, the effects of Cox5a knockdown on cell viability and the expression of cell death-related factors under hypoxic conditions were investigated. Cox5a knockdown was found to enhance cell death in H9c2 cells under both normoxic and hypoxic conditions (Fig. 5b). Subsequently, the expression of hypoxia-induced cell death-related factors was assessed via immunoblot analysis (Fig. 5c). First, Cox5a downregulation and hypoxia-induced factor 1α (Hif1α) upregulation by hypoxic stress were validated; then, significant increases in the levels of proapoptotic (Caspase 3, Cytochrome c, Caspase 9, Bak, and Bax), necroptotic (phosphorylated (p)-MLKL) and autophagic (LC3A/B and BNIP3) proteins and a marked decrease in the level of an antiapoptotic protein (Bcl-2) were observed in Cox5a-knockdown cells under normoxic and/or hypoxic conditions (Fig. 5c). These results show that Cox5a can inhibit cell death-associated signals induced by hypoxic stress.

### Discovery of miRs interacting with Cox5a in H9c2 cells

**Fig. 6:**
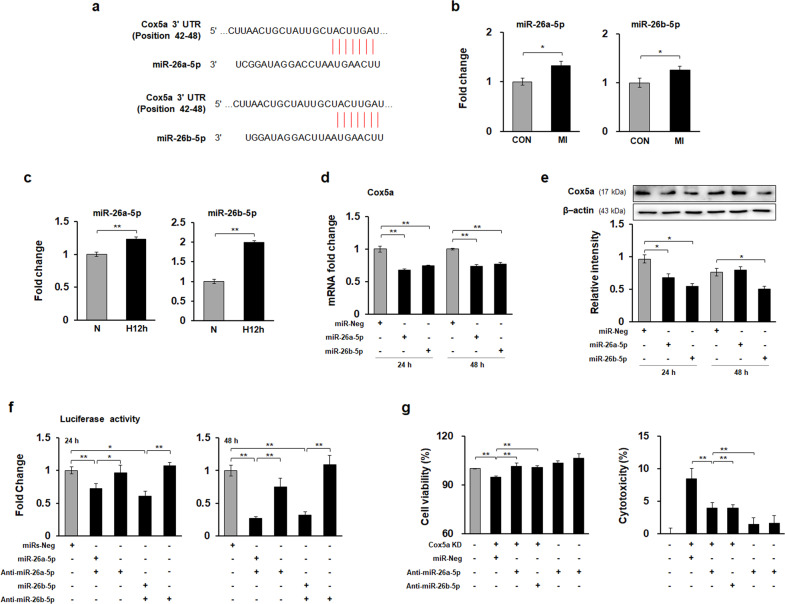
Identification of miRs targeting Cox5a to suppress hypoxic cell death. **a** Candidate miRs were identified using the TargetScan database. Expression of candidate miRs **b** in the hearts of control and MI rats and **c** H9c2 cells under normoxic and hypoxic conditions. The values are shown as normalized miR expression levels relative to U6 expression levels in triplicate samples. Changes in **d** the Cox5a transcript and **e** Cox5a protein expression levels induced by transfection of the candidate miRs were measured by qPCR analysis and immunoblot analysis, respectively. The qRT-PCR data are shown as the Cox5a expression level normalized to the Gapdh expression level in triplicate samples. Band intensities were measured as area densities and analyzed using ImageJ software. Relative intensity levels indicate the protein level normalized to the β-actin level. **f** Luciferase assay using the 3′UTR of Cox5a. **g** Changes in cell viability (left) and cytotoxicity (right) in H9c2 cells with Cox5a knockdown and miR treatment were determined by a WST-based cell viability assay and an LDH-based cytotoxicity assay, respectively. All data are representative of two independent experiments. Significant differences between groups were determined via ANOVA, with *p* values indicated as *p < 0.05 and **p < 0.01. CON control rat group, MI myocardial infarction rat group, N cells under normoxia, H12h cells exposed to hypoxia for 12 h, miRs-Neg negative control miRs, Cox5a KD Cox5a-knockdown cells.

We hypothesize that there are miRs that modulate Cox5a and thus may regulate hypoxia-induced cell death. In brief, candidate miRs controlling hypoxia-related cell death pathways were predicted and assessed using miR target prediction databases (TargetScan), and miR-26a/b-5p was identified to target Cox5a (Fig. 6a). The levels of these miRs in the hearts of MI rats (Fig. 6b) and hypoxic H9c2 cells (Fig. 6c) were significantly increased compared with those in the corresponding controls. To experimentally verify the interactions between the candidate miRs and Cox5a, H9c2 cells were transfected with the candidate miRs, and the transfected H9c2 cells showed significant decreases in both the Cox5a transcript (Fig. 6d) and Cox5a protein expression levels compared to the control cells (Fig. 6e). To further demonstrate that the candidate miRs directly target the 3′ UTR of Cox5a mRNA, we generated a luciferase reporter construct with the whole 3′ UTR of Cox5a, and H9c2 cells were transfected with this construct with/without the candidate miRs and/or their inhibitors (Fig. 6f). Cotransfection with the luciferase reporter construct and candidate miRs significantly inhibited luciferase expression, whereas treatment with candidate miR inhibitors restored luciferase expression in H9c2 cells (Fig. 6f). These results show that miR-26a/b-5p directly targets the 3′ UTR of Cox5a mRNA in a sequence-specific manner. To confirm miR-26a/p-mediated Cox5a regulation, cell viability and cytotoxicity were investigated in H9c2 cells after Cox5a knockdown and treatment with miR-26a/b-5p inhibitors (Fig. 6g). H9c2 cells were transfected first with Cox5a siRNA and then with each miR-26a/b-5p inhibitor 24 h later. After 24 h, cell viability and cytotoxicity were assessed. Cell death induced by Cox5a knockdown was abolished by miR-26a/b-5p suppression (Fig. 6g).

### Validation in primary cardiomyocytes

**Fig. 7:**
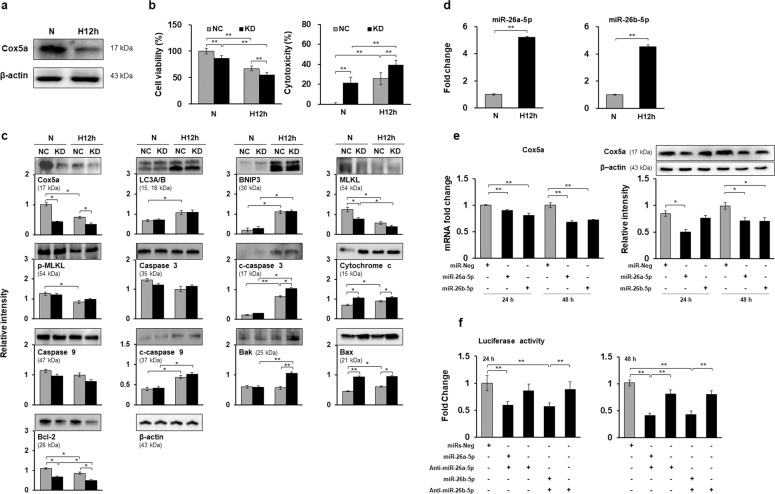
Validation of the expression and interaction of Cox5a and miR-26a/b-5p in primary cardiomyocytes. **a** Cox5a protein levels in primary cardiomyocytes under normoxic and hypoxic conditions. **b** Changes in cell viability (left) and cytotoxicity (right) in primary cardiomyocytes with Cox5a knockdown under normoxic and hypoxic stress. **c** Cox5a knockdown-induced expression of cell death-related factors. **d** Expression of miR-26a/b-5p in primary cardiomyocytes under normoxic and hypoxic conditions. **e** Changes in Cox5a transcript (left) and Cox5a protein (right) expression induced by miR-26a/b-5p treatment. **f** Luciferase assay using the 3′UTR of Cox5a. The qRT-PCR data are shown as the Cox5a and miR-26a/b-5p expression levels normalized to the Gapdh and U6 transcript levels, respectively, in triplicate samples. Band intensities were measured as area densities and analyzed using ImageJ software. Relative intensity levels indicate the protein level normalized to the β-actin level. Immunoblot experiments were performed twice independently. Significant differences between groups were determined via ANOVA, with *p* values indicated as *p < 0.05 and **p < 0.01. N cells under normoxia, H12h cells exposed to hypoxia for 12 h, NC negative control cells, KD Cox5a-knockdown cells, miRs-Neg negative control miRs.

To validate the effects of Cox5a on hypoxia-induced cell death and the direct association between Cox5a and miR-26a/b-5p, in vitro experiments were performed repeatedly in isolated primary cardiomyocytes. Cox5a protein expression was downregulated by hypoxic stress (Fig. 7a), and Cox5a knockdown promoted hypoxia-induced cell death (Fig. 7b) and the expression of apoptotic factors (Fig. 7c) in primary cardiomyocytes, as shown by the in vitro results. In addition, miR-26a/b-5p expression was elevated under low oxygen conditions (Fig. 7d), miR-26a/b-5p-transfected cells showed suppression of Cox5a expression (Fig. 7e), and miR-26a/b-5p directly interacted with Cox5a (Fig. 7f) in primary cardiomyocytes, similar to the findings in H9c2 cells. In summary, based on in vitro experiments using H9c2 cells and primary cardiomyocytes, Cox5a may affect hypoxia-induced cell death, and this pathway is closely related to miR-26a/b-5p.

## Discussion

As a terminal component of the electron transport chain, Cox enzymes, which are located in the inner mitochondrial membrane, catalyze the transfer of electrons from cytochrome c to oxygen^28,29^. The mammalian Cox family is composed of 13 monomers encoded by the mitochondrial (Cox1, Cox2, and Cox3) or nuclear (Cox4, Cox5a, Cox5b, Cox6a, Cox6b, Cox6c, Cox7a, Cox7b, Cox7c, and Cox8) genome that form dimers^28,29^. A lack of Cox activity has a considerable effect on energy metabolism and leads to substantial pathological conditions, such as Leigh syndrome, cardiomyopathy, lactic acidemia, and metabolic acidosis^30^. Moreover, the Cox5a transcript is abundant in heart muscle and skeletal muscle, whereas the Cox5a protein is distributed throughout various tissues, including the brain, kidneys, lungs, and endocrine tissue. Cox5a binds indirectly to Cox1 via Cox4 during Cox assembly^31^. Cox5a contributes to maintaining normal mitochondrial function, and abnormal expression of Cox5a affects Cox functions and mitochondrial dysfunction^32^.

Oxygen-regulated nuclear genes include hypoxic (Cox5b) and aerobic (Cox4, Cox5a, Cox7, and Cox8) genes^33^. Hypoxic genes are upregulated but aerobic genes are downregulated by exposure to anoxic or hypoxic stress^33^. Therefore, Cox5a expression is decreased in a low oxygen environment. Recent studies have shown that Cox5a overexpression inhibits cortical neurons during hypoxic-ischemic injury in rats^34^ and improves spatial recognition memory and hippocampal synaptic plasticity in mice^35^. In the heart, overexpression of Cox5a was found to protect against doxorubicin-induced cardiotoxicity in cardiomyocytes^31,36^. On the other hand, some reports have indicated an association between other Cox subunits and heart failure. Ischemic stress induces Cox3 upregulation, Cox1 downregulation, impaired Cox oxidative activity, and apoptosis in monkey models of MI^37^. In human myocardial insufficiency and dilated cardiomyopathy, decreased Cox4 results in impaired Cox oxidative activity^38^. Thus, several studies have reported associations between Cox5a and hypoxic environments and between other Cox subunits and heart disease. However, interestingly, there have been no reports of the functional correlation between Cox5a and hypoxia-induced heart disease.

In the present study, 22 proteins that showed significant differential expression between the hearts of control and MI rats were identified using proteomic analysis and 2-DE combined with MALDI-TOF-MS (Fig. 1b, d and Table 1). Network analysis showed that Cox5a has an interrelationship with most of the other 22 proteins (Fig. 1c). In addition, the RNA sequencing analysis results showed that Cox5a was downregulated in the hearts of MI rats compared with the hearts of control rats (Fig. 2). Downregulation of Cox5a was confirmed in vitro using H9c2 cells and primary cardiomyocytes under hypoxic conditions (Figs. 4 and 7). In summary, we found significantly decreased Cox5a expression in the hearts of MI rats, suggesting that Cox5a may contribute to the pathophysiology of ischemic heart disease.

Cell death can be induced via apoptosis, necrosis, autophagy, and necroptosis during MI, ischemia/reperfusion, and heart failure^4,39,40^. Pharmacological and genetic inhibition of cell death in these diseases reduces the infarct size and improves cardiac function^4^. Here, Cox5a knockdown in H9c2 cells under low oxygen conditions decreased cell viability and increased the expression of apoptotic, necroptotic, and autophagic proteins (Fig. 5). Moreover, Cox5a knockdown in primary cardiomyocytes isolated from neonatal rat hearts decreased cell viability, but affected the expression of only apoptotic factors under hypoxic conditions (Fig. 7). A recent study showed that Cox5a can protect H9c2 cells against anticancer drug-induced cell death^36^. Therefore, Cox5a may play a role in preventing cell death induced by hypoxic stress, although this activity depends on the cell type.

We hypothesized that miRs directly affect Cox5a in the MI model. If miRs are found to affect Cox5a, they can be used for MI prognosis determination, diagnosis, and/or treatment. Two miRs, miR-26a-5p and miR-26b-5p (miR-26a/b-5p), were identified using the TargetScan database (Fig. 6a). Moreover, these two miRs were found to directly affect Cox5a expression in H9c2 cells and primary cardiomyocytes in vitro (Figs. 6 and 7). miR-26a/b-5p plays diverse roles in various diseases and inhibits cell proliferation and aggressiveness in bladder cancer^41,42^. miR-26a protects vascular smooth muscle cells against H_2_O_2_-induced cell injury^43^ and increases autophagy to defend against ethanol-induced liver injury^44^. In addition, Han et al. identified certain negative autophagy regulators, including Cox5a, as miR-26a targets in human hepatoma cell lines^44^. The interaction between miR-26a-5p and Cox5a identified by these studies is the same as that observed in our present study, but the indications, mechanisms, and related factors are different. miR-26a prevents neuroinflammation in chronic sciatic nerve injury^45^, and miR-26b controls microglial inflammation in hypoxia/ischemia^46^. In addition, miR-26b suppresses liver fibrogenesis and angiogenesis by targeting PDGFR-β^47^.

The roles of miR-26a/b in cardiovascular disorders have been reported in recent studies. Circulating miR-26a-5p was found to be significantly increased in patients with CAD^48^. miR-26a inhibits the development of atherosclerosis by targeting TRPC3^49^, but enhances myocardial damage in myocardial ischemia and reperfusion^50^. miR-26a promotes myocardial fibrosis in acute MI^51^, and enhances autophagy activation in myocardial cells and cardiac hypertrophy by controlling GSK3β^52^. On the other hand, miR-26b relieves inflammation and remodeling in MI mice by suppressing the MAPK pathway^53^ and promotes the proliferation, survival, and angiogenesis of endothelial cells^54^. In the current study, we found that miR-26a/b-5p was significantly upregulated in the hearts of MI rats and hypoxic cardiomyocytes and that miR-26a/b-5p directly interacted with Cox5a in both H9c2 cells and primary cardiomyocytes under hypoxic conditions (Figs. 6 and 7). In conclusion, inhibition of Cox5a by miR-26 a/b-5p promotes hypoxia-induced cell death, suggesting that suppression of miR-26a/b-5p may prevent cell death induced by MI.

Although we are not the first to discover the interaction between Cox5a and miR-26a/b-5p, our current study provides convincing evidence supporting the negative roles of miR-26a/b-5p, the positive role of Cox5a, and their intercorrelation in MI rat models using multiomics analysis and functional studies. These results suggest that miR-26a/b-5p can be used for MI prognosis determination, diagnosis, and treatment. However, before miRs can be evaluated in clinical trials for MI, one problem must be overcome, namely, that miRs have diverse effects on various cells and diseases. Therefore, large animal studies and the accumulation of more data regarding these issues are required before miR therapeutics can be translated into clinical applications. Despite these limitations, miRs have incredible potential to become powerful tools to fight cardiovascular disease.

## Supplementary information

Supplementary Figure 1
