# Supplementary figures and images for: MicroRNA-26a/b-5p promotes myocardial infarction-induced cell death by downregulating cytochrome c oxidase 5a
Source: Exp Mol Med. 2021 Sep 13;53(9):1332–43. doi: 10.1038/s12276-021-00665-0 (PMC8492744; doi:10.1038/s12276-021-00665-0)

**Fig. S1.** Heatmap of the gene list for highlighted phenotypes in gene set enrichment analysis

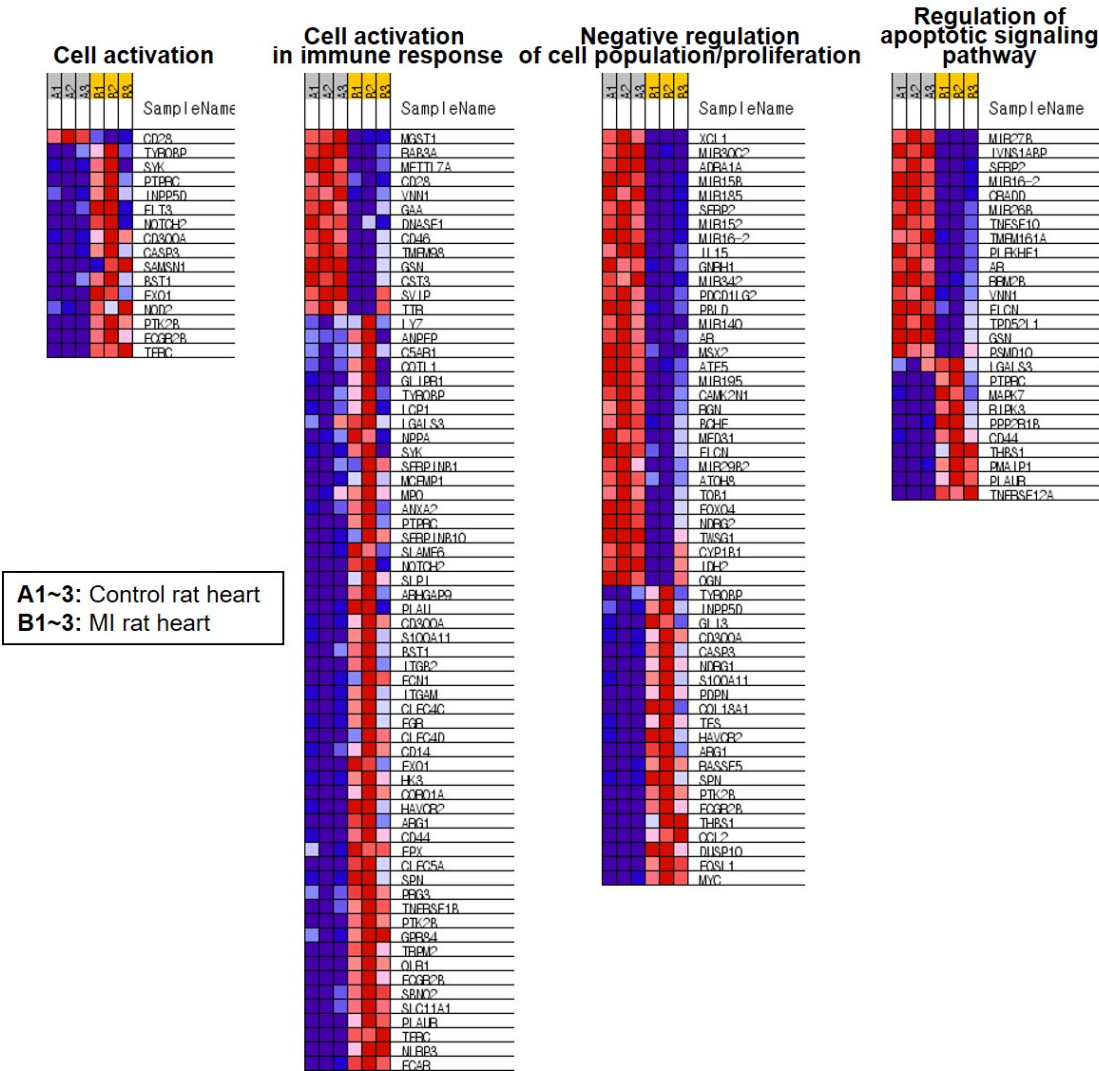

Supplement: Supplementary file 1 — Supplementary Figure 1 [file 12276_2021_665_MOESM1_ESM.pdf]
